# Descending thoracic aorta–abdominal aortic bypass and bilateral renal arterial blood circulation reconstruction are effective in atypical coarctation of the aorta with heart failure: a case report

**DOI:** 10.1186/s13019-021-01598-5

**Published:** 2021-08-04

**Authors:** Takuma Mikami, Takeshi Kamada, Toshiyuki Yano, Tomohiro Nakajima, Naomi Yasuda, Tsuyoshi Shibata, Keitaro Nakanishi, Ryo Harada, Syuichi Naraoka, Kojiro Toda, Nobutaka Nagano, Atsuko Muranaka, Nobuyoshi Kawaharada

**Affiliations:** 1 Department of Cardiovascular Surgery, Sapporo Medical University School of Medicine, 291, Minami 1-jo Nishi 16-chome Chuo-ku, Sapporo, 060-8543 Japan; 2 Department of Cardiovascular, Renal and Metabolic Medicine, Sapporo Medical University School of Medicine, Sapporo, Japan

**Keywords:** Atypical coarctation of the aorta, Anatomical bypass, Heart failure

## Abstract

**Background:**

There are a lot of reports of the renal failure and heart failure due to coarctation of the aorta. However, there are no case reports in which revascularization dramatically improved left ventricular function in patients with progressive decline in left ventricular function. Herein, we present a rare case in which the left ventricular function was dramatically improved by surgical treatment for progressive left ventricular dysfunction due to atypical coarctation of the aorta.

**Case presentation:**

A 58-year-old man underwent left axillary artery-bilateral femoral artery bypass at another hospital for atypical coarctation of the aorta due to Takayasu’s arteritis. Approximately 10 years later, he was re-hospitalized for heart failure, and the left ventricular ejection fraction gradually decreased to 28%. Computed tomography showed severe calcification and stenosis at the same site from the peripheral thoracic descending aorta to the lower abdominal aorta of the renal artery, and aortography showed delayed bilateral renal artery blood flow. An increase in plasma renin activity was also observed. Despite the administration of multiple antihypertensive drugs, blood pressure control was insufficient. We decided to perform surgical treatment to improve progressive cardiac dysfunction due to increased afterload and activated plasma renin activity. Descending thoracic aorta-abdominal aorta bypass and revascularization of the bilateral renal arteries via the great saphenous vein grafts were performed. Postoperative blood pressure control was improved, and the dose of antihypertensive drugs could be reduced. Plasma renin activity decreased, and transthoracic echocardiography 1.5 years later showed an improvement in contractility with a left ventricular ejection fraction of 58%.

**Conclusion:**

In atypical coarctation of the aorta in patients with decreased bilateral renal blood flow, heart failure due to renal hypertension, and progressive decrease in left ventricular contractility, descending thoracic aorta-abdominal aortic bypass and bilateral renal artery recirculation can be extremely effective.

## Background

Some reports have shown that axillary-bilateral femoral artery bypass is effective in cases of hypertensive heart failure due to atypical coarctation of the aorta [1, 2]. Axillary artery-femoral artery bypass has been reported to have good long-term results [3]; however, there are also reports of poor long-term results such as graft occlusion [4]. To the best of our knowledge, there are no reported cases that have required re-bypass treatment for atypical coarctation of the aorta in the long-term period after axillary-femoral artery bypass. In addition, although atypical coarctation of the aorta combined with hypertensive heart failure has been reported [1], there are no reports of cases requiring bilateral renal artery reconstruction by open surgery or improvement of decreased left ventricular contractility. Herein, we report a case of aortic stenosis due to atypical coarctation of the aorta in a patient who had undergone left axillary artery-bilateral femoral artery bypass grafting and repeated hypertensive heart failure in the postoperative period. He underwent open surgery of the descending thoracic aorta-abdominal aorta bypass and reconstruction of the bilateral renal artery. After surgery, the left ventricular contractility improved significantly.

## Case presentation

**Fig. 1:**
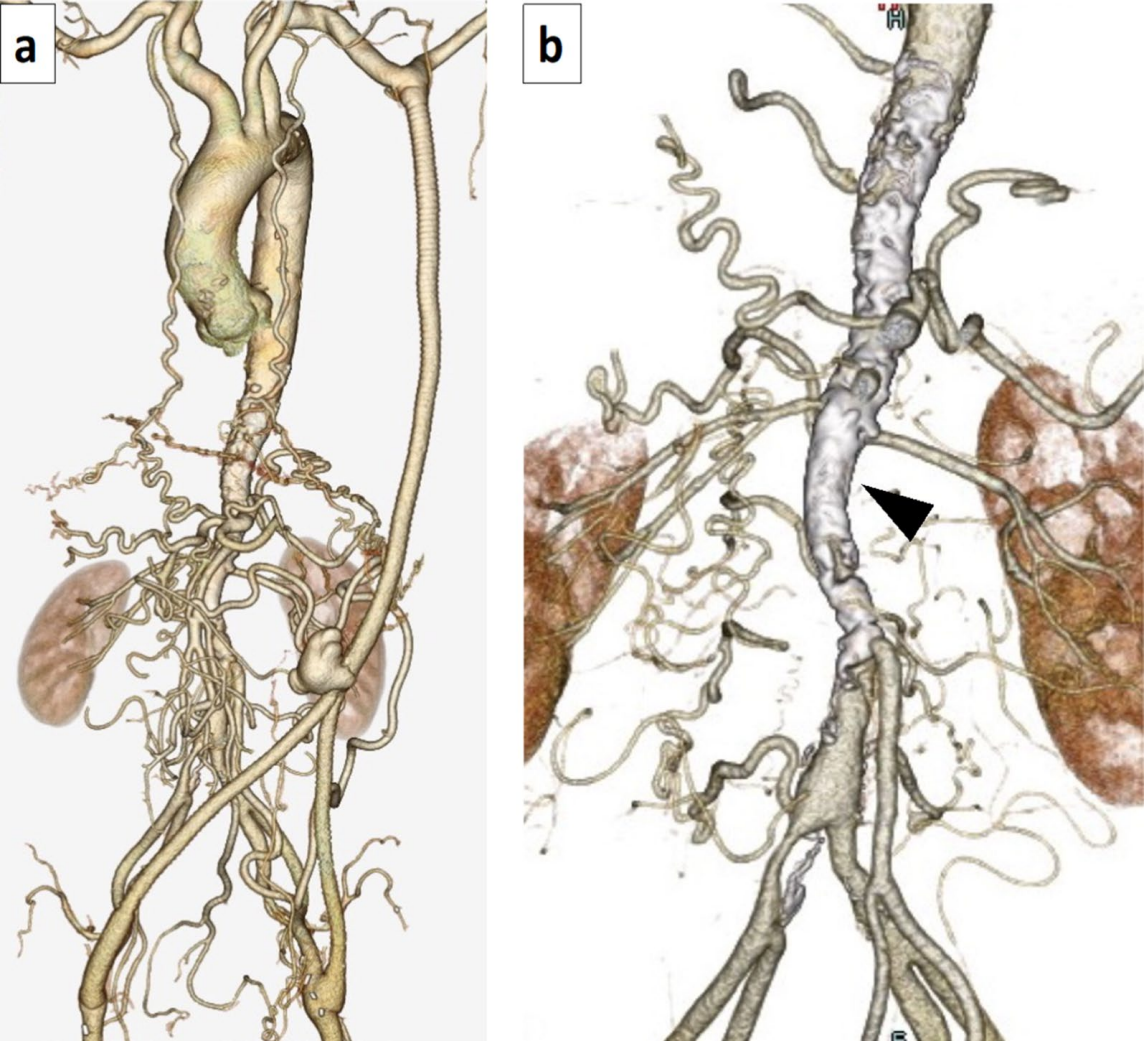
Preoperative computed tomography. **a** Preoperative 3D-CT showing the patent left axillary artery-bilateral common femoral artery bypass. Many collateral circulations to the lower extremity arteries are present. **b** 3D-CT of the thoracoabdominal aorta showing a high degree of calcification and stenosis around the descending aorta to the abdominal aorta (arrowhead)

**Fig. 2:**
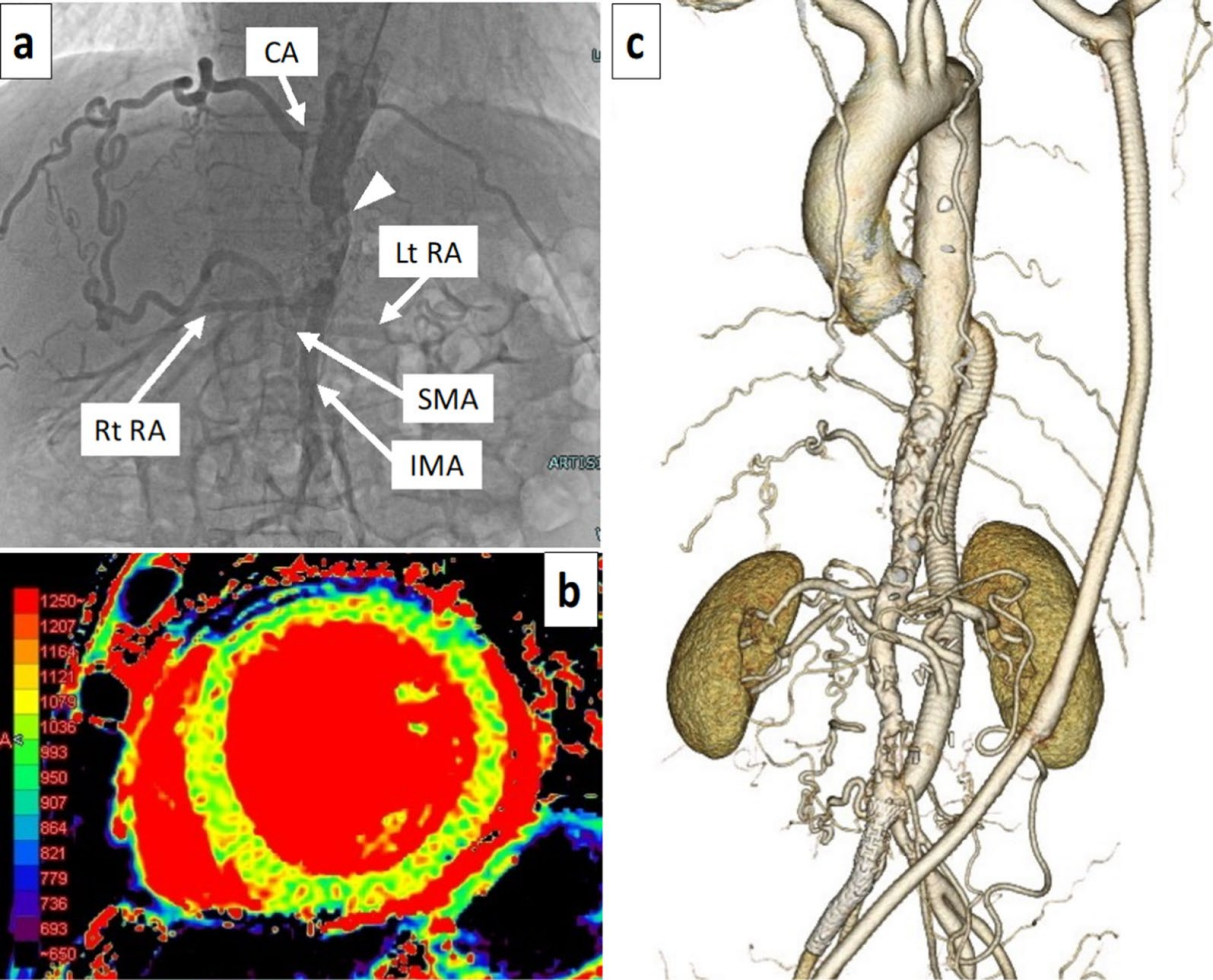
Preoperative aortography, magnetic resonance imaging, and postoperative computed tomography. **a** Aortography showing the superior mesenteric artery (SMA) imaged by the well-developed collateral circulation from the celiac artery (CA). Bilateral renal arteriography was delayed. Severe stenosis is observed in the epigastric aorta of the renal artery (arrowhead). **b** T1 mapping images on visceral MRI shows a slight increase in the T1 value of the left ventricular wall and no fibrosis progression on the left ventricular wall. **c** Postoperative 3D-CT showing that the descending thoracic aorta-abdominal aorta bypass and bilateral renal artery bypass were patent

A 58-year-old man previously underwent left axillary-bilateral femoral artery bypass grafting at another hospital for atypical coarctation due to Takayasu’s arteritis. He was re-hospitalized approximately 10 years later due to heart failure, and a progressive decrease in left ventricular contractility was observed, with the left ventricular ejection fraction (LVEF) decreasing from 54 to 28%. Although multiple antihypertensive drugs were administered, he was referred to our hospital to investigate the cause of the progressive decrease in left ventricular contractility and repeated heart failure due to poor blood pressure control. He had comorbidities, including pemphigoid. ^18^F-fluorodeoxyglucose (^18^F-FDG) positron emission tomography/computed tomography (PET-CT) performed at the previous hospital did not detect an accumulation of ^18^F-FDG in the aorta. His laboratory data were as follows: creatinine, 2.93 mg/dL; blood urea nitrogen, 65.7 mg/dL; estimated glomerular filtration rate, 19 mL/min/1.73 m^2^; C-reactive protein (CRP), 0.54 mg/dL; N-terminal pro-brain natriuretic peptide (NT-pro BNP), 17,014 pg/mL; aldosterone, 119 (reference value 36–240) pg/ml; and plasma renin activity, 10.6 (reference value 0.2–2.3) ng/ml/h. His blood pressure values were as follows: right upper limb, 168/108 mmHg; upper left limb, 169/99 mmHg; right lower limb, 143/98 mmHg; and left lower limb, 131/101 mmHg. The ankle brachial index (ABI) values of the right and left were 0.85 and 0.78, respectively. Preoperative transthoracic echocardiography showed a LVEF of 28%. There was no left ventricular asynergy, and the wall motion showed diffuse severe hypokinesis. Left ventricular wall thickness of the interventricular septum (IVS) and posterior wall (PW) were 13 mm and 12 mm, respectively. Left ventricular hypertrophy was also observed. The left ventricular end diastolic and systolic diameters (LVDd and LVDs) were 57.4 mm and 47.5 mm, respectively, and left ventricular enlargement was detected. No significant valvular dysfunction was observed. Contrast-enhanced CT showed a patent left axillary artery—both femoral artery bypass and collateral circulation to the lower extremity artery (Fig. 1a). Significant calcification was observed from the distal thoracic descending aorta to the abdominal aorta, and severe stenosis was suspected at the same site (Fig. 1b). Aortography showed severe stenosis of the aorta from below the celiac artery to the abdominal aorta below the renal artery. Blood flow in the superior mesenteric artery flowed from the well-developed collateral circulation from the celiac artery, and flow imaging of the bilateral renal arteries was delayed (Fig. 2a). Coronary angiography showed no abnormal findings, including at the entrance. Contrast-enhanced cardiac magnetic resonance imaging (MRI) revealed no apparent delayed contrast on the left ventricular wall, and T1 mapping images of cardiac MRI presented no significant increase in the T1 value on the left ventricular wall (1,050 ms by 1.5 T modified Look-Locker inversion recovery method; the average value of normal control at our hospital was 1,000 ms), and fibrosis of the left ventricular wall was considered mild (Fig. 2b). Although CRP was weakly positive, CT showed only severe calcification of the aorta, and PET-CT performed at the previous hospital did not show active inflammation in the aorta due to Takayasu’s arteritis. Therefore, we decided to perform open surgery for uncontrolled renal hypertension and a progressive decrease in left ventricular contractility due to decreased bilateral renal blood flow. To reliably reduce afterload, we performed descending thoracic aorta-abdominal aorta bypass using a large-diameter graft and revascularization of the bilateral renal arteries via the great saphenous vein grafts.

Thoracotomy was performed under general anesthesia via the left lower 7th intercostal space. Approach to the retroperitoneal space was performed along the left rectus abdominis muscle. A skin incision was made across the left axillary artery-both femoral artery bypass. To secure blood flow to the lower limbs during aortic clamping, surgery was performed without transection of the left axillary artery-both femoral artery bypass grafts. The retroperitoneal space was peeled off without incising the diaphragm to expose the abdominal aorta. In the retroperitoneal space, the dorsal side of the kidney was dissected, and the diaphragm was incised on the left side of the aortic hiatus to create a tunnel from the left thoracic cavity to the retroperitoneal space. After systemic heparinization, the descending thoracic aorta was clamped at the Th9 level, and proximal anastomosis was performed using a 12-mm woven graft (Intergard^®^, Maquet, Sunderland, UK). The anastomosed woven graft was guided to the retroperitoneal space through the tunnel of the diaphragm and the dorsal side of the left renal artery, and distal anastomosis was performed just above the abdominal aortic bifurcation. During this period, lower limb blood flow was maintained by the patent left axillary-bilateral femoral artery bypass. The anterior side of the left kidney was peeled off to expose the anterior surface of the abdominal aorta and the left and right renal arteries. The great saphenous vein was collected and anastomosed to the left and right renal arteries, and the vein graft stump was anastomosed to the woven graft. The left leg of the exposed left axillary artery-bilateral femoral artery bypass graft was resected just below the wound, considering the risk of postoperative infection. The operative time was 388 min.

**Fig. 3:**
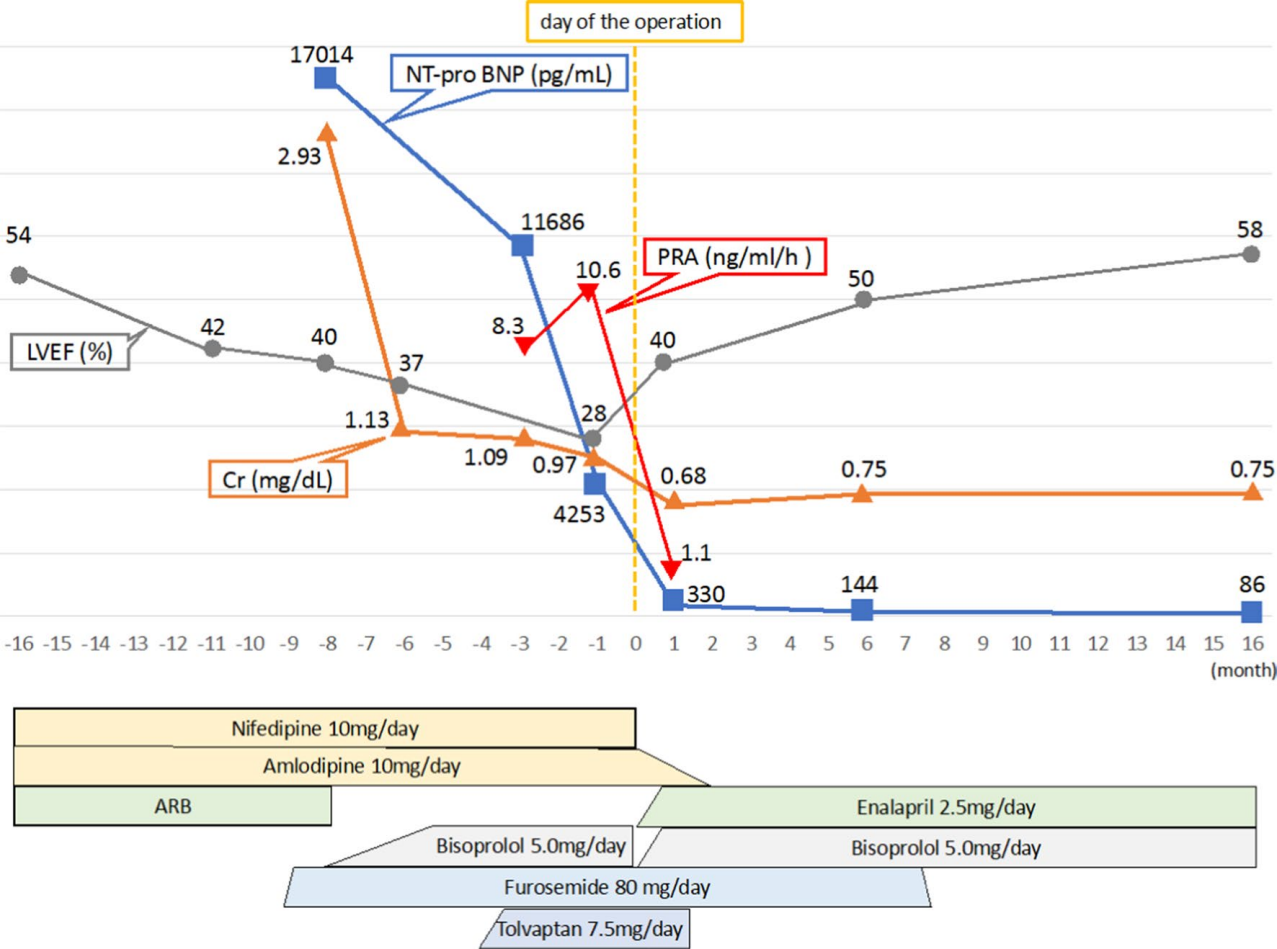
Pre- and postoperative progress. 0 month was the month of the surgery. Six months postoperatively, cardiac function improves to preoperative levels. NT-pro BNP and Cr tend to decrease with the control of heart failure, but a further decrease is observed postoperatively. PRA is high and tends to increase preoperatively. Postoperatively, it decreases. The dose of antihypertensive drugs can be reduced after the operation. In addition, diuretics used to control heart failure can be discontinued postoperatively. ARB, angiotensin II receptor blocker; Cr, creatinine; LVEF, left ventricular ejection fraction; NT pro BNP, N-terminal pro brain natriuretic peptide; PRA, plasma renin activity

No postoperative complications were observed. Postoperative 3D-CT showed that the bypass graft from the descending thoracic aorta to the abdomen ran parallel to the aorta on the dorsal side of the left renal artery and was patent. The great saphenous vein graft to the bilateral renal arteries was also patent (Fig. 2c). Postoperative ABI improved to 1.12 on the right and 1.06 on the left; the upper-limb blood pressure was improved to 128/91 mmHg with good control, and the dose of antihypertensive drugs could be reduced. He was discharged from the hospital 16 days after surgery. Blood test findings after discharge showed a decrease in NT-pro BNP level (78 pg/dL), aldosterone level (58 pg/mL), and plasma renin activity (1.1 ng/ml/h). Transthoracic echocardiography 1.5 years after surgery showed a marked improvement in LVEF of 58% (Fig. 3). Left ventricular wall thickness and left ventricular diameter decreased: IVS, 9.0 mm; PW, 8.7 mm; LVDd, 41.6 mm; and LVDs, 28.6 mm. Left ventricular reverse remodeling was performed.

Written informed consent was obtained from the patient to publish this case report and accompanying images.

## Discussion

Atypical coarctation of the aorta is defined as stenosis outside the aortic isthmus, in contrast to typical coarctation of the aorta, which has stenosis of the aortic isthmus. The causes include inflammatory vascular lesions (mainly Takayasu’s arteritis), arteriosclerosis, and congenital [5]. Hypertension in the upper body due to decreased renal blood flow and increased vascular resistance causes increased left ventricular afterload and serious complications, resulting in a poor prognosis [6].

In the present case, an axillary-femoral artery bypass had been performed approximately 10 years prior to rehospitalization; although the bypass was very patent, uncontrolled upper limb hypertension and heart failure due to increased left ventricular afterload were observed. The left ventricular contractility decreased progressively. In addition, plasma renin activity was high, and bilateral renal arteries showed delayed contrast enhancement in aortic findings, suggesting a decrease in bilateral renal artery blood flow. Open surgery was indicated because few findings were suggestive of active Takayasu’s arteritis. Surgical treatment methods, such as graft replacement, patch formation, bypass surgery, and stent placement [7], have been reported. Among them, the route of bypass surgery has been frequently discussed, and many reports have included aortic-aortic bypass or axillary-femoral artery bypass. Some case reports have presented axillary-femoral artery bypass that does not require thoracotomy or laparotomy and is effective with minimal invasiveness [1, 2]. However, reducing the afterload may be insufficient because it is bypassed by a small-diameter graft and because the perfusion to the abdominal organ becomes retrograde blood flow from the femoral artery. Poor long-term patency rate of grafts was also reported [4]. Aortic-aortic bypass can be used with large-diameter grafts, leading to reliable reduction of left ventricular afterload, and revascularization of abdominal branches is possible depending on the route. However, its drawback is the high level of surgical invasiveness because it involves thoracotomy and laparotomy. In addition, either the ascending aorta or descending aorta will be selected as the inflow of the bypass graft. There are various reports on the route and method [8–10], and each has advantages and disadvantages. When the ascending aorta is selected as the graft inflow, it is necessary to use a side clamp when anastomosing the ascending aorta or clamping the ascending aorta under a cardiac arrest with the establishment of the cardiopulmonary bypass. In either case, the burden on the heart is massive, especially in cases of severe cardiac dysfunction. In addition, the graft may become long and kinked, and some grafts may pass through the abdominal cavity, resulting in digestive tract complications. When the descending aorta is selected as the graft inflow, the graft length is shortened, and it is possible to revascularize the abdominal branch from the retroperitoneum, which is an advantage. However, aortic surgery with left thoracotomy requires separate lung ventilation, which is difficult in patients with severe respiratory dysfunction, and increases the risk of postoperative respiratory complications [11]. In this case, a reliable reduction in left ventricular afterload and improvement in bilateral renal artery perfusion are necessary. Therefore, surgery was selected for aortic-aortic bypass grafting with the left thoracotomy retroperitoneal approach and revascularization of the bilateral renal arteries using great saphenous vein graft. Simultaneous visual field of the bypassed graft and bilateral renal arteries is required for revascularization of the bilateral renal arteries. For this reason, when aorto-aorta bypassing, a tunnel was created through the aortic hiatus by the retroperitoneal approach from the dorsal side of the left kidney, and the ventral side of the left kidney was peeled off. It was possible to secure a simultaneous visual field and reconstruct the bilateral renal arteries.

Renal function and cardiac function are closely related, and the interaction between them is known as cardiorenal syndrome [12]. Hypertension and decreased renal blood flow are known to induce an increase in the renin–angiotensin–aldosterone (RAA) system and other specialized pathways. Fibrosis of the myocardium and kidneys has been reported to cause chronic and irreversible deterioration of cardiac and renal functions [13]. In this case, the plasma renin activity was predicted to increase, so the RAA system was enhanced due to the decrease in renal blood flow accompanying the progression of the coarctation of the aorta, and the increase in left ventricular afterload led to a progressive decrease in left ventricular contractility. Preoperative T1 mapping images by cardiac MRI showed a slight increase in the T1 value of the left ventricular wall. Therefore, fibrosis of the left ventricular wall was mild, and reverse remodeling of the left ventricle after surgery and improvement of left ventricular contractility could be expected. The reduction of afterload and the improvement of RAA system activation by the thoracic descending aorta-abdominal aorta bypass and the reconstruction of blood flow to the bilateral renal arteries led to an improvement in left ventricular function.

## Conclusion

We encountered a rare case of decreased bilateral renal blood flow and heart failure due to atypical coarctation of the aorta and progressive decrease in left ventricular contractility. Improvement in cardiac function was observed after thoracic descending aorta-abdominal aortic bypass and reconstruction of the bilateral renal artery.

## Data Availability

The datasets of the current study are available from the corresponding author upon reasonable request.

## Abbreviations

LVEF: Left ventricular ejection fraction
^18^F-FDG: ^18^F-fluorodeoxyglucose
PET-CT: Positron emission tomography/computed tomography
CRP: C-reactive protein
NT-pro BNP: N-terminal pro-brain natriuretic peptide
ABI: Ankle brachial index
IVS: Interventricular septum
PW: Posterior wall
LVDd and LVDs: Left ventricular end diastolic and systolic diameters
MRI: Magnetic resonance imaging
RAA: Renin–angiotensin–aldosterone

## References

[1] Ishizuka M, Yamada S, Maemura S, Yamamoto K, Takizawa M, Uozumi H (2017). Axillofemoral bypass markedly improved acute decompensated heart failure and kidney injury in a patient with severely calcified stenosis of thoracoabdominal aorta (atypical aortic coarctation). Int Heart J.

[2] Fukunaga N, Uryuhara K, Koyama T (2016). Axillobifemoral bypass for aortitis syndrome in a living-donor liver transplant patient. Ann Vasc Dis.

[3] Samson RH, Showalter DP, Lepore MR, Nair DG, Dorsay DA, Morales RE (2018). Improved patency after axillofemoral bypass for aortoiliac occlusive disease. J Vasc Surg.

[4] Schneider JR, McDaniel MD, Walsh DB, Zwolak RM, Cronenwett JL (1992). Axillofemoral bypass: outcome and hemodynamic results in high-risk patients. J Vasc Surg.

[5] Celoria GC, Patton RB (1959). Congenital absence of aortic arch. Am Heart J.

[6] Taketani T, Miyata T, Morota T, Takamoto S (2005). Surgical treatment of atypical aortic coarctation complicating Takayasu's arteritis-experience with 33 cases over 44 years. J Vasc Surg.

[7] Keith DS, Markey B, Schiedler M (2002). Successful long-term stenting of an atypical descending aortic coarctation. J Vasc Surg.

[8] Obata S, Mukai S, Morimoto H, Hiraoka T, Uchida H, Yamane Y (2013). Successful ascending aorta-abdominal aorta bypass graft through the left thoracic cavity in a patient with atypical coarctation. Ann Vasc Dis.

[9] Terada T, Yuasa T, Hasegawa M, Horiuchi K, Nakata S, Yasuura K (2014). Ascending to abdominal aorta extraanatomic bypass for descending aortic coarctation: a reconstruction technique without laparotomy or left thoracotomy. Ann Vasc Dis.

[10] Kim YS, Cho YH, Sung K, Kim DK, Chung S, Park TK (2020). Clinical outcome of extraanatomic bypass for midaortic syndrome caused by Takayasu Arteritis. Ann Thorac Surg.

[11] Etz CD, Zoli S, Kari FA, Mueller CS, Bodian CA, Di Luozzo G (2009). Redo lateral thoracotomy for reoperative descending and thoracoabdominal aortic repair: a consecutive series of 60 patients. Ann Thorac Surg.

[12] Zannad F, Rossignol P (2018). Cardiorenal syndrome revisited. Circulation.

[13] Rockey DC, Bell PD, Hill JA (2015). Fibrosis-a common pathway to organ injury and failure. N Engl J Med.

